# Mobile-based mindfulness meditation intervention’s impact on mental health among young male judo athletes in South Korea: a quasi-experimental study

**DOI:** 10.1038/s41598-024-63637-0

**Published:** 2024-06-03

**Authors:** Ye Hoon Lee, Weisheng Chiu, Juhee Hwang, Sihyeon Noh

**Affiliations:** 1 Division of Global Sport Industry, Hankuk University of Foreign Studies, Faculty Office Building 104, Yongin-si, Gyunggi-do 449791 Republic of Korea; 2 Lee Shau Kee School of Business and Administration, Hong Kong Metropolitan University, Hong Kong, China

**Keywords:** Anxiety, Depressive symptoms, Mind–body exercise, Resilience, Self-esteem, Stress, Quality of life, Psychology

## Abstract

Young athletes commonly encounter various mental health challenges due to the distinct pressures inherent in sports environments. This study investigates the effectiveness of mobile-delivered mindfulness meditation interventions in alleviating mental health indicators of depression, perceived stress, and anxiety, and enhancing self-esteem and resilience among young male judo athletes in South Korea. Pre- and post-test questionnaires were completed by 53 judo athletes. Participants were then allocated to the intervention group (N = 27; M_age_ = 13.77 [SD = 1.11]), which used a mobile meditation software program, or the control group (N = 27; M_age_ = 13.56 [SD = 1.05]). Data analysis compared intervention and control group scores using multiple statistical methods, including independent sample t-tests, paired sample t-tests, and 2 (time) × 2 (group) repeated measures analysis of variance. Following the intervention, the mindfulness group exhibited significant enhancements in the mental health indicators of depression (GMD = 2.74 [95% CI 0.90–4.56], Cohen’s D = 0.84), perceived stress (GMD = 0.35 [95% CI 0.002–0.70], Cohen’s D = 0.56), and anxiety (GMD = 0.2 [95% CI 0.001–0.40, Cohen’s D = 0.56]. Self-esteem also had a significant increase (GMD = 0.55 [95% CI − 0.22 to − 0.88], Cohen’s D = 0.95). The findings of this study underscore the potential benefits of mobile-delivered mindfulness meditation interventions in addressing mental health challenges among young male judo athletes. The significant enhancements observed in scores on measures of depression, perceived stress, anxiety, and self-esteem among participants in the mindfulness group highlight the effectiveness of such interventions in promoting mental health in sports settings.

## Introduction

The most commonly seen mental health difficulties among adolescents include depression, stress, and anxiety disorders^1,2^. It is important to acknowledge that a considerable proportion (20%) of children and adolescents have reported experiencing notable mental health challenges, such as depression and anxiety, in the course of their educational journey^3,4^. South Korea is one of many countries in which depression is common among young people. According to data from the National Health Insurance Service^5^, the number of children and adolescents who received hospital treatment for depression increased by 18.9% from 30,536 in 2019 to 39,870 in 2021. In just the first half of 2022, there were 33,990 cases, nearly approaching the total for the previous year. The number of individuals diagnosed with anxiety disorders also surged from 16,797 in 2019 to 23,593 in 2021. Unfortunately, the presence of depression, stress, and anxiety in adolescents can have significant and wide-ranging negative effects across multiple aspects of their lives. For example, adolescents who encounter elevated levels of such symptoms may manifest enduring feelings of sorrow, heightened irritability, alterations in sleep patterns, and challenges in maintaining focus^6,7^. These manifestations have the potential to impede their capacity to effectively participate in academic pursuits and extracurricular activities, which in turn has the potential to result in academic underperformance, reduced self-esteem, and a heightened likelihood of discontinuing one’s education^8,9^. Importantly, if not properly addressed, these symptoms have the potential to endure throughout an individual’s adult life and could potentially exacerbate more serious mental health conditions^2,10^.

In the culture of competitive sports, young athletes are held to a standard emphasizing unwavering commitment, personal sacrifice, and a readiness to push their physical limits for the enhancement of their individual performance and their team^11,12^. Taking this code to an extreme is regarded as emblematic of a genuine athlete, earning the acceptance and respect of peers who recognize him or her as part of the team^13^. However, it is crucial to acknowledge that these endeavors can profoundly affect the overall well-being of young athletes^14^. In fact, young athletes may face a range of stressors, such as excessive training, demands from coaches, expectations from peers and family, team obligations, fear of failure, or the loss of their athletic identity resulting from injury or retirement^11,15–17^. Of particular concern is the rising prevalence of mental health issues among young athletes, which prompts us to contemplate their holistic development^12,18^. Rates of depression among young athletes have been found to vary from 15.6 to 23.7%^19,20^, indicating that they are susceptible to experiencing clinically significant mental health concerns.

Young athletes’ struggles with excessive levels of physical, emotional, psychological, and academic pressures may also negatively impact their self-esteem and resilience. Self-esteem, defined as the personal evaluation of one’s own value, worth, and capabilities^21^, and resilience, the ability to adapt and maintain psychological well-being in challenging situations^22^, are two important psychosocial factors that can significantly impact young athletes’ performance, confidence, social interactions, adaptability in competition, and even overall development^23–25^. Thus, both self-esteem and resilience serve as foundations of young athletes’ healthy development, not only in terms of their athletic pursuits but also in the development of their character and future achievements. More importantly, resilience and high self-esteem can help protect them from mental illness^26,27^. Considering the negative implications of the stresses on young athletes, it is imperative to acknowledge and offer prompt treatments for mental health issues in young athletes in order to avert enduring negative outcomes and promote their comprehensive well-being.

Unfortunately, young athletes face several significant challenges when it comes to receiving mental health treatment, largely influenced by sport culture and other factors. In many sport environments, there is a prevailing stigma surrounding mental health issues, and there is an emphasis on physical toughness, perfectionistic mindsets, and resilience^28^. This atmosphere can give rise to fears among athletes that they might be perceived as weak if they seek help for their mental health challenges, ultimately leading to a reluctance to openly discuss their struggles^29,30^. The persistent pressure to excel and deliver peak performances looms large, and admitting to mental health issues can be viewed as an excuse for any perceived dip in performance, further discouraging athletes from reaching out for assistance. This apprehension about potential exclusion from their sport can compel athletes to remain silent about their symptoms^31^. Additionally, the rigorous training regimens, routines, and dietary habits designed to optimize their performance can effectively mask underlying mental health concerns, while balancing the demands of academics and athletics, along with limited time, can also deter athletes from seeking treatment^32,33^. In some cases, there is a lack of awareness and education about mental health signs and symptoms, leaving young athletes without early intervention and support^29^. The prevailing but inaccurate belief that athletes are immune to mental health issues, coupled with the scarcity of research focusing on this specific demographic, further obstructs their access to suitable treatment. While engaging in sports might positively impact mood, adhering to a structured sports regimen does not necessarily shield young athletes from the possibility of having mental health concerns and symptoms associated with them.

Mindfulness-based meditation interventions, which refer to interventions that primarily focus on developing attentional skills and qualities of present-moment awareness to enhance different dimensions of an individual's wellbeing^34^, may present a promising approach for addressing the mental health difficulties experienced by young athletes. Mindfulness techniques possess the capacity to alleviate the adverse effects of mental health concerns on this susceptible demographic by fostering self-awareness, emotional control, and stress management. Rumination, characterized by repetitive and intrusive thoughts focused on negative experiences or emotions, is a key cognitive process implicated in the maintenance of depression and anxiety^35^. By shifting individuals’ focus from ruminative thinking to present-moment awareness, mindfulness meditation has been shown to alleviate symptoms of depression and stress^35^. In recent years, implementation of mindfulness-based meditation interventions within educational institutions with the aim of addressing depression, stress, and anxiety among adolescents has become much more common^36^. Multiple experimental studies have investigated the impact of mindfulness meditation on collegiate athletes’ mental well-being^37–41^. For example, Rooks et al.^41^ examined the impact of four weeks of mindfulness training on emotional well-being in college football players. The mindfulness training used in this study focused on its relevance to football-specific challenges, such as managing distractions during performance and controlling emotional reactions. Various mindfulness exercises, including mindfulness of breathing, body scan, and awareness exercises, were introduced to the athletes. The results showed that increased engagement in mindfulness training was associated with better-sustained attention, reductions in anxiety, and increased positive affect. Similarly, Jones et al.^37^ investigated the impact of an eight-week mindfulness training program on psychological well-being, sleep, athletic coping skills, and rowing performance in collegiate rowers. The results demonstrated that the mindfulness group exhibited enhancements in psychological well-being, including reduced depression and anxiety, compared to the control group. More recently, Myall et al.^42^ conducted a systematic review and meta-analysis on the impact of mindfulness meditation on elite adolescent and adult athletes, revealing significant positive effects on mental health outcomes. Despite these findings, research has thus far primarily focused on adult elite athletes, leaving a gap in the literature regarding young athletes. Moreover, previous studies often employed quite lengthy mindfulness training sessions lasting 45 to 150 minutes^42^ In contrast, our interventions were designed to last approximately 15 minutes, accommodating the busy schedules of young athletes engaged in rigorous training regimens.

Mobile-based mindfulness meditation programs offer a flexible strategy for enhancing mental health. They successfully remove obstacles that could discourage individuals from adopting mindfulness meditation by offering easily available and handy tools for practicing it through smartphones and tablets^43^. In comparison to face-to-face programs that may be perceived as daunting, mobile applications present a reduced threshold for participation, enabling users to commence their mindfulness journey at a self-determined pace^44,45^. Thus, these applications ensure reliable access to mindfulness meditation, regardless of the availability of classes or athletes’ often grueling travel schedules. Moreover, with the integration of configurable elements, mobile applications can offer a wide range of programs that cater to individual interests or mental health requirements, hence potentially enhancing the efficacy of therapies^46^. In addition, these platforms incorporate interactive elements, progress monitoring, and reminders, which enhance motivation and compliance^47^. Finally, it is worth noting that these services demonstrate cost-effectiveness, often providing low or no-cost alternatives, thus significantly enhancing accessibility^48^. Certain mobile applications also promote community engagement by incorporating such elements as discussion forums and professional coaching, thereby facilitating support and motivation for users in their pursuit of mindfulness. Although mobile-based mindfulness meditation programs have considerable advantages in addressing overall well-being, there is little research exploring their impact within the sport context. Thus, in the study we report herein, we sought to examine the potential impacts of mobile-based mindfulness meditation programs, which offer numerous advantages, on the outcomes of young athletes.

Within the demanding realm of high-performance sports culture, where young athletes are held to standards emphasizing commitment and physical prowess, the necessity of mindfulness meditation interventions to address mental health issues among young athletes becomes increasingly apparent. These athletes, while striving for excellence, often encounter a multitude of stress-inducing factors such as excessive training, high performance expectations, and fear of failure. As evidenced by substantial rates of depression among young athletes, it's clear that mental health concerns are prevalent within this demographic, underscoring the urgency for intervention. Moreover, the intersection of physical, emotional, and academic pressures can detrimentally impact crucial psychosocial resources such as self-esteem and resilience, vital for overall well-being and performance. Yet, despite the evident need for support, young athletes encounter significant barriers to accessing mental health treatment, perpetuated by stigma, misconceptions, and a lack of awareness within sports environments. Given these challenges, it is imperative to integrate mindfulness meditation interventions tailored to the unique needs of young athletes, not only to alleviate immediate concerns but also to foster a culture of holistic well-being essential for their comprehensive development and success.

The overall objective of this study is to examine the effectiveness of mindfulness meditation interventions, administered via mobile platforms, in alleviating mental health indicators of depression, perceived stress, and anxiety and the psychological well-being of self-esteem and resilience among young athletes. We hypothesized that the participants in the mindfulness group would exhibit significant reductions in indicators of mental health problems and significant improvements in psychological well-being indicators after the intervention compared to the control group. Through an in-depth exploration of the benefits of mindfulness applications for athletes, we aim to shed light on the untapped potential of these programs for enhancing the well-being of young athletes.

## Results

### Participants

**Figure 1:**
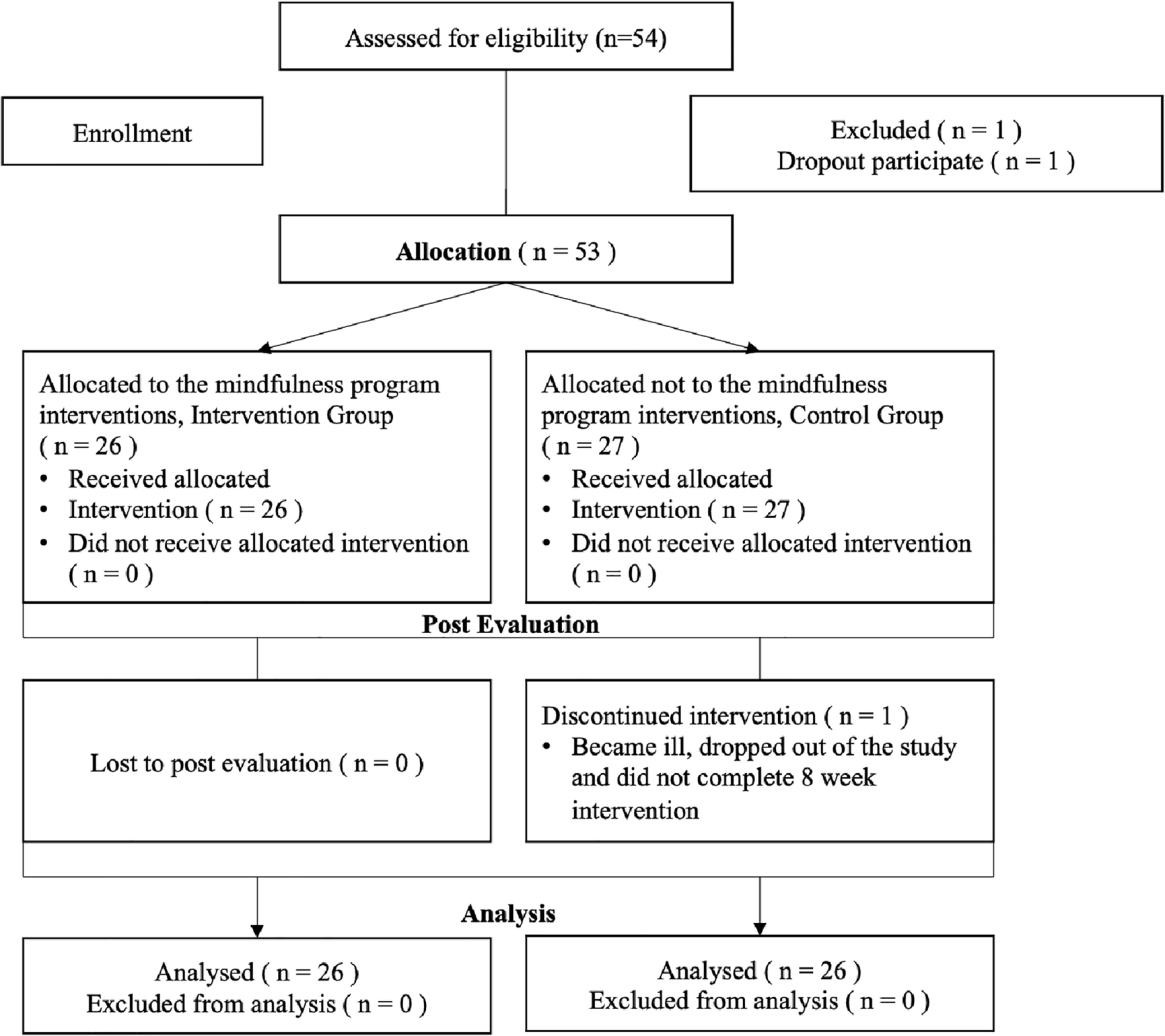
CONSORT flowchart.

Figure 1 displays the CONSORT (Consolidated Standards of Reporting Trials) diagram, which graphically illustrates the movement of participants during the study. Among the original 53 participants, 26 individuals (49.05%) were assigned to the treatment group, whereas 27 individuals (50.95%) were allocated to the control group. Significantly, all 26 participants in the mindfulness group and 26 out of 27 participants (96.29%) in the control group successfully completed the post-evaluation. Hence, a total of 52 participants were included in the final data analysis. Comprehensive demographic information is presented in Table 1.

**Table 1:** Demographic characteristics.

Characteristics	Mindfulness group	Control group	*p*
	*N* = 26	*N* = 27	
Categorical variables, N (%)			
Gender			
Male	26 (100%)	27 (100%)	
Experience in mobile-based mindfulness app			
No	26 (100%)	26 (96.3%)	
Continuous variables, M ± SD			
Age	13.77 ± 1.11	13.56 ± 1.05	0.47
Height (cm)	169.17 ± 9.82	167.25 ± 7.14	0.42
Weight (kg)	73.96 ± 22.27	64.70 ± 14.04	0.08
Judo experience (month)	25.23 ± 16.37	35.03 ± 22.35	0.09
Internet use (min)	213.07 ± 59.24	225.18 ± 99.39	0.59
Internet self-efficacy	3.20 ± 0.95	3.51 ± 0.70	0.17

### Results from the baseline- and post-test between the two groups

The baseline characteristics of the individuals in the mindfulness group (*n* = 27) and control group (*n* = 27) are presented in Table 2. The findings indicated that the mindfulness group displayed elevated levels of depression (*M* = 5.50, *SD* = 3.89), anxiety (*M* = 0.43, *SD* = 0.45), perceived stress (*M* = 1.95, *SD* = 0.74), negative affectivity (*M* = 1.51, *SD* = 1.35) in comparison to control group (*M* = 4.22, *SD* = 3.74 for depression; *M* = 0.28, *SD* = 0.33 for anxiety; *M* = 1.61, *SD* = 0.54 for perceived stress; *M* = 1.36, *SD* = 0.57 for negative affectivity). In the context of psychological well-being, the mindfulness group had lower levels of self-esteem (*M* = 3.44, *SD* = 0.64) and resilience (*M* = 2.94, *SD* = 0.82) compared to the control group (*M* = 3.81, *SD* = 0.72 for self-esteem; *M* = 3.45, *SD* = 1.73 for resilience). Crucially, there were no statistically significant disparities among the two groups in terms of any of the factors at the beginning of the study.

**Table 2:** Mean differences between mindfulness group and control group at baseline- and post evaluation.

	Baseline				Post			
	Mindfulness group	Control group	GMD (95% CI)	Cohen’s D	Mindfulness group	Control group	GMD (95% CI)	Cohen’s D
Depression	5.50 (3.89)	4.22 (3.74)	1.28 (− 0.82 to 3.38)	0.34	2.76 (2.53)	3.65 (4.10)	− 0.89 (− 2.78 to 1.01)	0.26
Perceived stress	1.95 (0.74)	1.61 (0.54)	0.34 (− 0.01 to 0.69)	0.52	1.60 (0.49)	1.67 (0.57)	− 0.07 (− 0.37 to 0.22)	0.13
Anxiety	0.43 (0.46)	0.28 (0.33)	0.15 (− 0.06 to 0.37)	0.37	0.23 (0.22)	0.30 (0.40)	− 0.07 (− 0.25 to 0.10)	0.22
Negative affectivity	1.51 (0.51)	1.35 (0.57)	0.16 (− 0.13 to 0.46)	0.29	1.29 (0.36)	1.48 (0.66)	− 0.19 (− 0.48 to 0.10)	0.35
Self-esteem	3.47 (0.64)	4.02 (0.51)	− 0.54 (− 0.87 to − 0.22)*	0.95	3.81 (0.67)	3.68 (0.89)	0.13 (− 0.30 to 0.57)	0.17
Resilience	2.94 (0.82)	3.28 (0.92)	− 0.33 (− 0.82 to 0.15)	0.39	3.45 (1.73)	3.23 (1.10)	0.22 (− 0.57 to 1.03)	0.15

Table 2 also provides a summary of the comparisons between the two groups after the intervention. The control group exhibited significantly higher mean scores of depression (*M* = 3.65, *SD* = 4.10), anxiety (*M* = 0.31, *SD* = 0.40), perceived stress (*M* = 1.67, *SD* = 0.57), negative affectivity (*M* = 1.48, *SD* = 0.66) in comparison to control group (*M* = 2.76, *SD* = 2.53 for depression; *M* = 0.23, *SD* = 0.22 for anxiety; *M* = 1.60, *SD* = 0.49 for perceived stress; *M* = 1.29, *SD* = 0.36 for negative affectivity). In the context of psychosocial dimensions, the experiment group had higher levels of self-esteem (*M* = 4.02, *SD* = 0.51) and resilience (*M* = 3.28, *SD* = 0.92) compared to the control group (*M* = 3.68, *SD* = 0.89 for self-esteem; *M* = 3.23, *SD* = 1.10 for resilience). Again, there was no statistically significant difference between the groups for all the variables.

Next, paired sample t-tests were undertaken to determine if there were significant differences in the levels of depression, anxiety, perceived stress, self-esteem, and resilience depending on the utilization of a mobile-based mindfulness meditation program across time (Table 3). The findings for depression indicated a reduction in the average score from 5.50 ± 3.89 to 2.76 ± 2.53 for the mindfulness group (Group mean difference [GMD] = 2.74 [95% CI 0.90–4.56], Cohen’s D = 0.34). Regarding anxiety and perceived stress, the mindfulness group also exhibited a significant decrease from 0.43 ± 0.45 to 0.23 ± 0.22 for anxiety (GMD = 0.2 [95% CI = 0.001 to 0.40], Cohen’s D = 0.37) and 1.95 ± 0.74 to 1.60 ± 0.49 for perceived stress (GMD = 0.35 [95% CI 0.002–0.70], Cohen’s D = 0.52). There was also a significant difference in self-esteem (GMD = 0.53 [95% CI − 0.88 to − 0.22], Cohen’s D = 0.52) while there was no significant difference in negative affectivity (GMD = 0.23 [95% CI − 0.02 to 0.47], Cohen’s D = 0.50) and resilience (GMD = 0.34 [95% CI − 0.82 to 0.15], Cohen’s D = 0.38) in mindfulness group. For control group, there was no statistical difference between pre- and post-test for all the variables.

**Table 3:** Mean differences between baseline- and post evaluation for both groups.

	Mindfulness group				Control group			
	Baseline	Post	GMD (95% CI)	Cohen’s D	Baseline	Post	GMD (95% CI)	Cohen’s D
Depression	5.50 (3.89)	2.76 (2.53)	2.73 (0.90 to 4.56)*	0.84	4.22 (3.74)	3.65 (4.10)	0.56 (− 1.59 to 2.73)	0.15
Perceived stress	1.95 (0.74)	1.60 (0.49)	0.35 (0.002 to 0.70)*	0.56	1.61 (0.54)	1.67 (0.57)	0.15 (− 0.37 to 0.24)	0.11
Anxiety	0.43 (0.45)	0.23 (0.22)	0.20 (0.001 to 0.40)*	0.56	0.28 (0.33)	0.30 (0.40)	− 0.02 (− 0.23 to 0.17)	0.05
Negative affectivity	1.51 (0.51)	1.29 (0.36)	0.23 (− 0.02 to 0.47)	0.49	1.35 (0.57)	1.48 (0.66)	− 0.12 (− 0.47 to 0.21)	0.21
Self-esteem	3.47 (0.64)	4.02 (0.51)	− 0.54 (− 0.87 to − 0.22)*	0.95	3.81 (0.67)	3.68 (0.89)	0.13 (− 0.30 to 0.57)	0.17
Resilience	2.94 (0.82)	3.28 (0.92)	− 0.33 (− 0.82 to 0.15)	0.39	3.45 (1.73)	3.23 (1.10)	0.22 (− 0.57 to 1.03)	0.15

*p < 0.05.

### Effectiveness of mobile-based mindfulness meditation program on the outcomes

**Figure 2:**
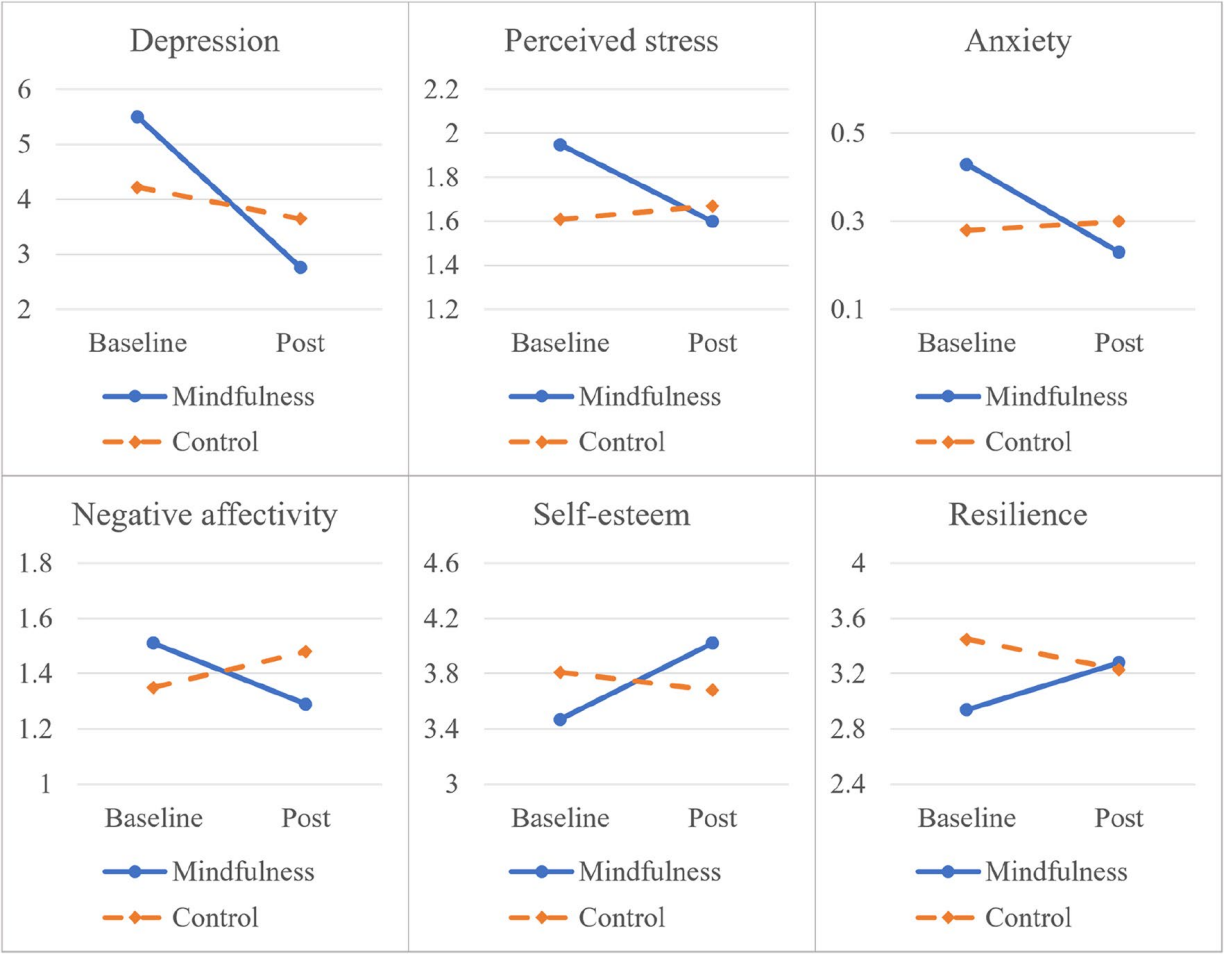
Results of the interactional analysis.

In the 2 (group) x 2 (time) RMANOVA, there were significant main impacts of time on the depression scores (*F*_1, 50_ = 5.50, *p* = 0.023, η_p_^2^ = 0.10) and self-esteem (*F*_1, 50_ = 9.60, *p* = 0.034, η_p_^2^ = 0.09). However, there was no significant main effects of time on anxiety (*F*_1, 50_ = 2.42, *p* = 0.12, η_p_^2^ = 0.05), perceived, stress (*F*_1, 50_ = 2.11, *p* = 0.15, η_p_^2^ = 0.04), negative affectivity (*F*_1, 50_ = 0.39, *p* = 0.53, η_p_^2^ = 0.01), and resilience (*F*_1, 50_ = 0.01, *p* = 0.940, η_p_^2^ = 0.00). Further, there were significant interaction effects between time and group for depression (*F*_1, 50_ = 4.15, *p* = 0.047, η_p_^2^ = 0.08); perceived stress (*F*_1, 50_ = 4.66, *p* = 0.036, η_p_^2^ = 0.09); negative affectivity (*F*_1, 50_ = 4.59, *p* = 0.037, η_p_^2^ = 0.08); and self-esteem (*F*_1, 50_ = 15.97, *p* = 0.000, η_p_^2^ = 0.24). Nevertheless, there was no significant interaction impacts between group and time found for anxiety (*F*_1, 50_ = 3.35, *p* = 0.07, η_p_^2^= 0.06) and resilience (*F*_1, 50_ = 3.65, *p* = 0.06, η_p_^2^ = 0.07). These results are depicted in Fig. 2.

## Discussion

This study investigated the effectiveness of mobile-based mindfulness meditation programs for improving mental health indicators (such as depression, perceived stress, and anxiety) and psychological well-being indicators (such as self-esteem and resilience) among male judo athletes in South Korea. Following the eight-week intervention, the mindfulness group showed a significant improvement in depression and self-esteem when compared to the control group. We also found that there were interaction effects between group and time for the variables of depression, perceived stress, and self-esteem. Our results demonstrate the utility of including mobile-based mindfulness meditation programs for young athletes in a competitive sporting context.

The notable decrease in depression and perceived stress among young athletes after the mobile-based mindfulness meditation intervention corresponds with abundant data from children^65,66^ and young athletes^36–38,41^. Mindfulness practices may equip young athletes with depression management and stress management techniques by emphasizing present-moment awareness and non-judgmental acceptance. Additionally, by learning to focus on the task at hand and accept their thoughts and emotions without judgment^67^, athletes may experience reduced levels of cortisol, a stress hormone, thereby mitigating the physiological impact of stress. This reduction in physiological stress responses can contribute to decreased feelings of anxiety among young athletes.

A common problem for athletes, especially in competitive sports, is rumination over past mistakes or excessive worry about future performances^29^. Mindfulness interventions promote present-focused attention, reducing the tendency to dwell on negative thoughts or overthink^34^. This shift from ruminative thinking to present-moment awareness can alleviate symptoms of depression and stress by breaking the cycle of negative thought patterns. Theoretically, the findings of this study underscore the potential of mobile-based mindfulness meditation programs as a viable intervention strategy for addressing mental health concerns among young athletes. The reluctance of young athletes to seek help due to fear of being perceived as weak or the fear of negative repercussions for their athletic careers necessitates the development of innovative, accessible, and stigma-free interventions^13^. Hence, the present study provides valuable insights into the potential efficacy of mobile-based mindfulness meditation programs as a discreet and flexible solution that circumvents some of the barriers hindering traditional face-to-face interventions.

The improvements in self-esteem within the mindfulness group emphasize the holistic nature of the intervention. Elevated self-esteem is pivotal in fostering coping mechanisms among young athletes facing mental health challenges, potentially acting as a protective factor against future issues. For young athletes, whose identities are often intertwined with their athletic performance^13^, self-esteem can be heavily influenced by both their achievements and the pressures associated with high-performance sports culture. Mindfulness activities foster heightened self-awareness, enabling individuals to impartially observe their thoughts, feelings, and sensations^67^. By developing a heightened sense of self-awareness, young athletes may be able to identify and confront negative self-perceptions or self-criticisms that may impact their self-esteem, instead of fixating on previous mistakes and future anxieties. Furthermore, by increasing their awareness of their internal experiences and decreasing the inclination to dwell on previous errors^68^, individuals can cultivate a more empathetic and tolerant mindset towards themselves, which in turn promotes the development of a positive self-perception and enhanced self-worth. Additionally, on a physiological level, previous studies indicate that engaging in mindfulness techniques might cause alterations in the brain's structure, specifically in regions linked to self-awareness and the management of emotions, such as the prefrontal cortex and the insula^69,70^. These brain modifications might influence the way individuals perceive themselves and promote a more favorable self-image, ultimately leading to enhanced self-esteem. Finally, mindfulness promotes the practice of constructive self-dialogue and the cultivation of a mentality focused on personal progress^71^. Young athletes who participate in competitive sports may develop a more stable and positive self-concept, leading to higher self-esteem, by embracing a mindset that prioritizes effort, growth, and resilience rather than focusing solely on outcomes.

Despite our finding of improvements in other psychological areas, such as self-esteem, there was not a significant changes in negative affectivity and resilience among young athletes after the intervention. This is contrary to what we initially expected. This discovery prompts thought-provoking questions about the subtle impacts of mindfulness meditation programs on particular facets of psychological well-being. Resilience, defined as the capacity of an individual to adjust and recover from difficult situations^22^, continues to be a crucial element in successfully overcoming the obstacles encountered by young athletes. Although mindfulness practices often focus on developing resilience by improving coping strategies and emotional regulation, the absence of significant enhancement in resilience among our study participants necessitates more investigation. Recognizing unforeseen discoveries in research is crucial. In this instance, the lack of notable enhancement in resilience, despite other favorable psychological transformations, suggests that further research will be needed to achieve deeper understanding of how mindfulness meditation interventions specifically influence various aspects of mental well-being. Future research that investigates the intricacies of enhancing resilience in mindfulness meditation programs, potentially examining factors such as the length, substance, or methods of delivering interventions that may more effectively promote resilience among young athletes, is needed.

## Managerial implications

The findings of this study have several practical implications. First, the demonstrated reduction in symptoms of depression, anxiety, and perceived stress among the mindfulness group underscores the potential of integrating mobile mindfulness meditation interventions into the support systems for young athletes. This suggests that incorporating accessible and flexible mindfulness meditation programs into the daily routines of young athletes can serve as a proactive measure to address mental health challenges commonly experienced in competitive sports environments^65^. Therefore, coaches and athletic departments can consider implementing tailored mobile mindfulness applications or integrating existing platforms into the training and support frameworks for young athletes, thereby promoting holistic well-being and mental resilience.

Second, our results demonstrate the accessibility and adaptability of mobile mindfulness applications, providing actionable insights for real-world implementation. The reduced participation threshold and individuals' ability to commence their mindfulness journey at a self-determined pace through mobile applications align with the need for flexible and personalized mental health support in competitive sports settings. Therefore, athletic departments and coaches can consider incorporating configurable elements and progress monitoring features in mobile mindfulness applications tailored for young athletes^66^. For example, it may incorporate interactive elements, reminders, and community engagement features, enhancing motivation, compliance, and support networks and ultimately fostering a culture of mental well-being and resilience within athletic communities.

Finally, the findings of this study suggest the importance of developing educational and training initiatives aimed at raising awareness about the benefits of mindfulness and promoting its adoption among young athletes. Coaches, trainers, and sports educators can leverage the findings of this study to incorporate mindfulness training into overall athletic development programs, fostering a culture of mental resilience and well-being within sports communities^67^. Doing so can help young athletes manage their mental health, build their emotional resilience, and enhance their overall performance both on and off the field.

## Limitations and future research directions

Although this study shows encouraging results, it is important to take into account certain limitations. First, utilizing self-report tools to evaluate psychological variables may create subjective biases. Although these tools are frequently used, depending solely on self-reported data may not comprehensively reflect the intricacies of mental health. By including multi-method assessments or clinician-rated evaluations, a more thorough understanding of the intervention's impact can be achieved.

A primary limitation of this study is the use of a quasi-experimental design, wherein the intervention group and control group were not randomly assigned. Instead, one school volunteered to implement meditation practices, while the other school served as the control group. The lack of randomization in group assignment may result in differences between the intervention and control groups beyond the intended intervention, such as variations in student demographics, school culture, or external factors. These confounding variables could impact the observed outcomes and limit the generalizability of the findings. Future research involving randomized control trials so as to enhance internal validity and establish causal relationships between the mindfulness meditation intervention and outcomes is needed.

Another significant limitation of this research is the omission of performance metrics in assessing the impact of mindfulness meditation interventions on young athletes. While our study focused on evaluating the effects of mindfulness meditation on mental health outcomes, the absence of performance measures limits the comprehensiveness of our findings. In future research endeavors, it is imperative to address this limitation by incorporating comprehensive performance metrics alongside assessments of mental health outcomes. This would allow for a more nuanced understanding of the interplay between mindfulness meditation interventions, mental well-being, and athletic performance among young athletes. On a different note, future studies could integrate prosocial measures of compassion, empathy, and tolerance into the assessment protocol. By examining the impact of mindfulness meditation interventions on these additional variables, researchers can gain a more comprehensive understanding of the intervention's effects on promoting prosocial attitudes and behaviors among young athletes.

Furthermore, the study specifically focused on young athletes who were actively participating in judo sports. Examining the possible effects of mobile mindfulness meditation programs in various sports, age groups, and levels of competition could be useful for developing customized therapies for specific subgroups of young athletes. An investigation of the correlation between these elements and the effectiveness of mindfulness meditation programs could provide a more detailed understanding of how the intervention affects different demographic and athletic groups. Therefore, future studies should be designed to include a wider range of participants from various sports and diverse demographic backgrounds.

Finally, to allocate participants into the experimental and control groups, this study employed a convenience sampling method, where participants were categorized into groups based on their availability and consent to participate. While convenience sampling allowed for the recruitment of participants from the targeted population, it is important to note that this method may not ensure randomization and could potentially introduce selection biases. Future research endeavors should consider alternative methods of participant allocation, such as randomization, to enhance the rigor and validity of our study design.

## Conclusion

Previous research indicates a significant requirement for customized therapies due to the high occurrence of mental health difficulties among young athletes. The distinctive challenges experienced by this group, such as the demands of a competitive sports culture, academic expectations, and the pervasive negative perception of mental health in sports settings, add to the challenges of addressing their psychological welfare. Curiously, the general public is recommended to participate in physical activity as a treatment for mental health issues. However, competitive athletes partake in intense physical activity, which renders the intervention less suitable. In light of this paradoxical circumstance, we implemented the mindfulness meditation program as a means of addressing the mental challenges faced by young athletes. Notably, the significant reductions in depression, perceived stress, and anxiety, along with the significant enhancement in self-esteem within the mindfulness group after the intervention highlight the potential of mobile-based mindfulness meditation training in effectively addressing mental health concerns among young athletes. By reducing the obstacles to seeking assistance and providing a versatile and easily available method of intervention, these programs have considerable promise for fostering the overall well-being of this susceptible group.

## Methods

### Study design and population

This study employed a pretest-posttest nonequivalent control group quasi-experiment design. The participants were selected through convenience sampling, mainly from two middle school judo teams. The recruitment period spanned from June 2023 until August 2023. The lead investigator reached out to several middle schools to inquire about their willingness to take part in the experiment. Two schools demonstrated interest and were chosen, and a total of 53 young athletes were asked to participate. The participants were categorized into two groups: one school served as the mindfulness group (N = 26), which participated in mindfulness meditation sessions (referred to as the mindfulness group), while the other group served as the control group (N = 27). The mindfulness group used mobile-based programs that encompassed four distinct modules: meditation instruction, stress management, anxiety alleviation, and self-esteem improvement. Each module consisted of a range of 3–7 sessions. The control group did not receive any targeted intervention. Athletes were eligible for inclusion in the study if they were young athletes who are actively engaged in athletic activities and willing to take part in the study and comply with the intervention protocol, including attending mindfulness sessions and completing assessments. Additionally, they could not have had prior experience with formal mindfulness or meditation practices. Finally, individuals with a history of diagnosed mental health disorders, such as depression or anxiety disorders, were excluded to maintain focus on preventive interventions rather than clinical treatment, and participants with physical limitations or medical conditions that might restrict their participation in athletic activities or mindfulness exercises were excluded for safety and feasibility reasons.

### Sample size calculation

Determination of the suitable sample size for this study was conducted using the G*Power calculator^49^. The aim of the study was to achieve a significance level of 0.05 and a statistical power of 95%. This was done by aiming for an effect size of 0.29, a figure that was obtained from prior research that investigated the influence of mobile mindfulness interventions on depression^50^. Based on the calculations conducted, it was estimated that a minimum sample size of 24 participants was necessary in order to attain the requisite statistical power. In order to address the dropout rate of 57% observed in computer-based psychological treatment^51^, the total sample size was revised to include 38 participants while maintaining a power of 0.95. As a result, it was expected that the incorporation of 53 participants would guarantee sufficient statistical power for the present investigation.

### Ethical concern

The study was granted ethical approval by the Hankuk University of Foreign Studies Institutional Review Board (IRB) under the reference HIRB-202310-HR-001. Prior to commencing the study, all necessary authorizations were obtained to assure adherence to ethical standards and protect the rights of the participants. Potential participants were explicitly informed about the study's goals and procedures, and only those who willingly provided written informed consent were included in the research.

### Instruments

The study encompassed surveys of mental health and psychological well-being. The survey included questions about participants' demographic information, duration of internet usage, and level of confidence in internet use, as well as three mental health questionnaires (i.e., depression, perceived stress, and anxiety) and two psychological well-being questionnaires (i.e., self-esteem and resilience).

#### Depression

The evaluation of depression was conducted utilizing the Korean Patient Health Questionnaire-9 (PHQ-9). The PHQ-9 is a dependable and accurate instrument for evaluating and diagnosing depression symptoms^52^. This comprehensive assessment consists of nine questions that evaluate signs of depression, such as diminished interest or pleasure, mood fluctuations, disruptions in sleep patterns, exhaustion, and alterations in eating habits. Participants are required to assign a rating to each item on a scale of 0 (indicating not at all) to 3 (indicating nearly every day). A greater PHQ-9 total score signifies a higher number of depression symptoms, whereas a lower score implies a smaller number. The Cronbach's alpha coefficient of our study sample was 0.76, while the validation study showed a coefficient of 0.81^52^.

#### Perceived stress

We measured stress using the Korean Brief Encounter Psychosocial Instrument (BEPSI-K). The dynamic interaction model of stress and health impacts underpins this instrument^53^. Research has established that the BEPSI-K, the Korean version of the BEPSI, is reliable and valid^54^. This five-part questionnaire examines stress related to discouragement, unmet basic needs, and uncertainty about the future. The response rating scale spans from 1 (not at all) to 5 (always). Higher BEPSI-K values indicate more stress, whereas lower numbers indicate less stress. In this investigation, our sample's Cronbach's alpha coefficient was 0.78, which is highly similar to the validation study's 0.95^54^.

#### Anxiety

The 7-item Generalized Anxiety Disorder Scale assessed GAD symptoms. Its simplicity and limited number of elements make this device suited for primary care^55^. The scale lists anxiety symptoms including restlessness, impaired stress management, increased worry, and hypervigilance. Participants are asked to rate their frequency of experiencing each issue from 0 (not at all) to 3 (near every day). High GAD-7 scores indicate frequent occurrence and high intensity of generalized anxiety disorder symptoms, while low scores indicate lower levels of symptoms. Our sample's Cronbach's alpha coefficient was 0.79. This matches the validation study's 0.92 Cronbach's alpha^56^.

#### Negative affectivity

We employed the abbreviated versions of Negative Affectivity Scale of Positive Affectivity and Negative Affectivity Scale (PANAS) as developed by Watson et al.^57^. to assess the negative affectivity of the young athletes. The 5-point scale spanned from 1 (indicating very slightly or not at all) to 5 (indicating extremely). The items encompassed terms that represented various forms of negative affectivity such as fear and distress. The Cronbach's alpha coefficient for our sample was 0.83 and 0.93 in the previous study^58^.

#### Self-esteem

To measure the participants’ levels of self-esteem, the Rosenberg Self-Esteem Scale (RSE)^59^ was used. It consists of a total of 10 items, with 5 positive and 5 negative statements. The negative items were reverse-scored, so higher total scores indicate higher levels of self-esteem. The items include statements such as “I think I have a lot of good qualities” and “I believe I can do things as well as most people.” Responses were on a 4-point Likert scale ranging from “strongly disagree” (1) to “strongly agree” (4). Higher total scores indicate a higher level of self-esteem. The reliability coefficient (Cronbach’s α) is 0.78 in previous studies^60^, and it was 0.85 in this study.

#### Resilience

The 10-item version of the Connor-Davidson Resilience Scale^61^ (CD-RISC 10) was utilized in the present study. While the original CD-RISC^22^ has shown inconsistency in its factor structure across various studies, as noted by Lee et al.^62^, the CD-RISC 10 has consistently exhibited a stable and unidimensional factor structure in multiple studies^63,64^. Participants provided their responses to the 10 items by utilizing a five-point Likert-type scale spanning from 1 (indicating “strongly disagree”) to 5 (indicating “strongly agree”). Illustrative examples include statements such as "I possess the capacity to adjust in response to changes" and "experiencing and managing stress can enhance my resilience." In the current investigation, the internal consistency reliability was found to be good, with a Cronbach's α of 0.73 and it was 0.84 in the previous literature^62^.

### Procedure

The paper-pencil survey was conducted twice, during Week 0 and Week 8. One of the researchers visited each school and administered the questionnaires in person. In order to facilitate a seamless survey procedure, the researcher gave instructions to the participants and the coaches prior to conducting the survey.

Following their training sessions, participants engaged in mindfulness sessions under the guidance of a trained coach who could provide support and guidance as needed. These sessions used the mindfulness meditation programs offered by the commercial mindfulness mobile application. They participated in sessions lasting roughly 15 minutes, three times a week for 8 weeks, following a predetermined timetable. Consequently, participants completed a total of 24 mindfulness sessions. The researchers monitored participants' mindfulness participation by utilizing the commercial mobile app. Specifically, using the mobile mindfulness app, researchers were able to monitor the participants' advancement in their meditation sessions. In addition, the coach took and submitted photographs of participants’ mindfulness sessions to enhance the monitoring process via the app. The researcher documented the mindfulness logs obtained from the application and the session photographs in a collaborative document. While the experimental group received the mindfulness meditation interventions, the control group remained active through participation in their regular judo exercise routines, without engaging in the meditation program.

In considering the possible adverse effects of the meditation program, several measures were implemented to prioritize participant safety and well-being. First, participants were provided with detailed information about the nature of the intervention and given the opportunity to ask questions or express concerns before consenting to participate. Throughout the intervention period, close monitoring of participants' well-being and responses to the mindfulness sessions was conducted by the coach of the mindfulness group. Any signs of discomfort or adverse reactions were documented and addressed promptly by the researchers, with participants having the option to withdraw from the study or discontinue participation at any time. Adherence to ethical guidelines ensured that principles of beneficence and non-maleficence were upheld, with any adverse effects observed being addressed in accordance with ethical standards.

### Mobile mindfulness meditation intervention

We used the Calm app for research purposes to implement a mobile mindfulness meditation program for participants. The application offers a variety of mindfulness meditation programs that allow users to choose topics that correspond with their goals and participate in customized mindfulness sessions. In this app, users can access personalized programs from among a large collection of guided mindfulness programs, specifically designed to target stress management, self-confidence augmentation, concentration enhancement, and depression relief. Thus, the research team developed a specialized curriculum specifically designed for young athletes who normally do not practice meditation. The curriculum commenced with comprehensive instruction in mindfulness exercises, aimed at familiarizing participants with foundational mindfulness techniques. These exercises included mindfulness of breath, body scan meditation, and mindful movement practices such as walking meditation. Participants were guided through these exercises to cultivate present-moment awareness, attentional focus, and non-judgmental acceptance of their thoughts, emotions, and bodily sensations.

As participants progressed through the intervention, the curriculum advanced to incorporate material centered around stress management, anxiety reduction, and the enhancement of self-esteem. Stress management techniques introduced included mindfulness-based stress reduction (MBSR) exercises, such as body-awareness techniques and progressive muscle relaxation, aimed at reducing physiological arousal and promoting relaxation responses in the face of stressors. Additionally, cognitive restructuring exercises were integrated to help participants identify and challenge maladaptive thought patterns contributing to stress and anxiety. Additionally, anxiety reduction strategies included mindfulness-based cognitive behavioral techniques, such as mindfulness of thoughts and emotions, designed to increase awareness and acceptance of anxious thoughts and feelings without becoming entangled in them. Participants were taught skills to observe and detach from anxious thoughts, allowing for greater emotional regulation and decreased reactivity to anxiety-provoking situations. Finally, to address self-esteem improvement, the intervention incorporated mindfulness-based self-compassion practices aimed at fostering kindness, acceptance, and understanding toward oneself. Participants engaged in loving-kindness meditation exercises, cultivating feelings of warmth and compassion toward themselves and others. Additionally, cognitive techniques were employed to challenge self-critical thoughts and promote a more compassionate and balanced self-view.

Overall, the intervention provided a structured and progressive framework for developing mindfulness skills and applying them to manage stress, reduce anxiety, and enhance self-esteem among young athletes. By integrating mindfulness practices with evidence-based strategies from cognitive behavioral therapy, the curriculum aimed to empower participants with practical tools for promoting psychological well-being and resilience in the context of their athletic pursuits.

### Data analysis

Initially, frequency and descriptive analysis was employed to compute the frequencies and percentages of categorical variables, as well as the means and standard deviations (SDs) of continuous variables. Subsequently, a series of analyses was conducted to analyze the discrepancies between the baseline and post evaluations across different groups. First, the methodology employed an independent sample t-test to examine the differences in the proposed variables between the two groups at both baseline and post evaluation. Second, paired sample t-tests were conducted within each group to independently compare measurements taken at the beginning and after the evaluation. Subsequently, a 2 (group) × 2 (time) repeated measures analysis of variance (RMANOVA), where baseline and post-evaluation data were considered as dependent factors and the group was treated as the independent variable, was conducted.

### Ethical approval statement

All procedures performed in studies involving human participants were in accordance with the ethical standards of the institutional research committee.

### Informed consent

Informed consent was obtained from all individual participants included in the study.

## Data Availability

The datasets generated during and/or analyzed during the current study are available from the corresponding author on reasonable request.
